# Faith in the future of sport: value differences among young athletes in Finland, Italy, Poland, and Sweden

**DOI:** 10.3389/fpsyg.2026.1917306

**Published:** 2026-08-21

**Authors:** Faten Hamdi, Matteo De Angelis, Alexios Kolitsopoulos, Mariusz Granosik, Maurizio Bertollo

**Affiliations:** 1 High Institute of Sport and Physical Education, University of Jendouba, El Kef, Tunisia; 2 Department of Medicine and Aging Sciences, University “G. d'Annunzio, ”Chieti/Pescara, Italy; 3 Faculty of Educational Science, University of Lodz, Lodz, Poland; 4 Behavioral Imaging and Neural Dynamics Center (BIND), University of Chieti-Pescara “G. d'Annunzio, ” Chieti, Italy

**Keywords:** adolescent, Europe, sports, values, youth

## Abstract

**Introduction:**

Sports and physical activities play a key role in young people's physical, social, and moral development. However, the outcomes of this development may vary according to several factors, including age, gender, nationality, years of practice (YOP), and training tier, defined by athletes' training and performance levels. Therefore, this study aimed to examine how young athletes' values differ across countries and according to age, gender, training tier, and YOP.

**Methods:**

Within the framework of the European project “Faith in the future of sports”, the Youth Sport Values Questionnaire-2 (YSVQ-2), a 13-item survey categorizing values into Moral, Status, and Competence domains, was administered across four countries (Finland, Italy, Poland, Sweden).

**Results:**

A MANOVA conducted on a sample of 381 participants across the four European countries revealed statistically significant differences across all three value dimensions (Status, Competence, and Moral) and training tiers, age, gender and country.

**Discussion:**

Overall, the findings highlight subgroup variations in value endorsement, indicating that cross-national mean differences are moderated by training tier, age, and gender.

## 1 Introduction

Sport is a value-socialization area in which young people learn and negotiate moral priorities through rules, relationships, and the meanings attached to success and failure. Values are commonly defined as beliefs centered on one's self-concept and reflect the desire to reach goals and personal standards (Ring et al., 2023).

According to the universal theory of basic human values formulated by Schwartz (2012), human motivation is organized around 10 distinct values recognized across diverse cultural contexts. These values act as the cognitive standards underpinning individual attitudes, functioning as evaluative criteria for determining whether an object, person, or behavior is desirable or undesirable. Crucially, cross-cultural evidence demonstrates a surprising global consensus regarding the hierarchical priority of these values. This shared baseline is functionally imperative, as it provides the normative framework necessary to maintain social stability and coordinate collective behavior against disruptive individual

impulses (Schwartz, 2012). However, when translating this macro-framework into the specific domain of sport, contemporary literature suggests a crucial conceptual refinement. In line with recent cross-national investigations among European athletes (Petróczi et al., 2025), sport values are purposefully distinguished from rigid, trans-situational goals. In youth sport, three value types are especially salient: Moral, Competence and Status which align with Schwartz's domains (“Universalism,” “Self-direction,” and “Power”) and can serve as antecedents of athletes' attitudes (Lee et al., 2008). Lee et al. (2008) developed the Youth Sport Value Questionnaire-2 (YSVQ-2) aimed to measure those three value domains, that is, Moral, Competence and Status. Moral values have been associated with fairness, helpfulness and obedience, Competence values have been linked to achievement, showing skills, and finally Status values are associated with winning superiority, leadership, and public image (Ring et al., 2023). The survey has been translated and used in many countries and found good cross-cultural validity (Danioni et al., 2017).

Specifically, Moral values have been consistently associated with fairness and helpfulness, while Competence values are linked to show a skill and achievement (Ring et al., 2023). Conversely, Status values are characterized by a focus on winning and superiority (Ring et al., 2023). Cross-cultural adaptations of the YSVQ-2 have confirmed its psychometric robustness across various European nations (Danioni et al., 2017). Furthermore, cross-national investigations have begun to explore how macro-contexts affect value profiles; for instance, differences have been documented regarding the emphasis placed on playing “clean” or prioritizing enjoyment among diverse European cohorts (Woolway et al., 2021). While previous research has often examined these values through the lens of micro socializations, such as the role of the coach (Petróczi et al., 2025), there remains a critical gap in understanding how wider national and institutional frameworks systematically relate to the intensity of values. According to the universal theory of basic human values formulated by Schwartz (2012), human motivation is organized around ten distinct values recognized across diverse cultural contexts. These values act as the cognitive standards underpinning individual attitudes, functioning as evaluative criteria for determining whether an object, person, or behavior is desirable or undesirable. Crucially, cross-cultural evidence demonstrates a surprising global consensus regarding the hierarchical priority of these values. This shared baseline is functionally imperative, as it provides the normative framework necessary to maintain social stability and coordinate collective behavior against disruptive individual impulses (Schwartz, 2012). However, when translating this macro-framework into the specific domain of youth sport, contemporary literature suggests a crucial conceptual refinement. In line with recent cross-national investigations among European athletes (Petróczi et al., 2025), sport values are purposefully distinguished from rigid, trans-situational goals. Instead, drawing upon the operationalized notion of value structures (Schwartz, 1996), they are conceptualized as contextually rated belief sets shaped by the athletic environment.

In Europe, these institutional systems exhibit profound structural and socio-cultural diversity. Northern European systems share a foundational commitment to democratic access, public welfare, and operational decentralization (Bergsgard, 2025). This structural organization tends to prioritize personal growth and community cohesion over commercial or result-oriented pressures. Conversely, Southern European nations, such as Italy, often display distinct socio-cultural configurations, where social life and core values are frequently anchored around familial structures and relational prestige rather than a predominantly civic-oriented framework (Bergsgard, 2025). Theoretically, this contrast between a highly institutionalized, welfare-oriented civic system and a culture closely aligned with relational status and competitive achievement may modulate the athletic environment, potentially underpinning cross-national variations in athletes value endorsement. Because national sports systems operate through various intermediate factors, such as coaching behavior and team climate, we frame macro-level governance as a contextual background within which athlete value endorsement occurs, rather than a direct causal driver.

Previous studies already have explored values differences among European athletes but there was no consideration regarding age and experience of the athletes. The present study was conducted within the framework of the FAITH Erasmus+ project, a transnational initiative involving Italy, Sweden, Finland, and Poland, aimed at examining value differences among young athletes across nations and sport categories as a foundation for future teaching programs. Trainers should be aware of the differences between values in the athletes context given their association with sport behaviors. We expected to find differences in value endorsement scores (Moral, Status, and Competence) comparing cultural contexts and individual characteristics among young athletes across the four nations.

## 2 Methods

The YSVQ-2 was administered to youth and young adults across sports clubs and athletic facilities in four European countries: Italy, Sweden, Poland, and Finland, using a snowball sampling method. The study was approved by the Institutional Review Board of Psychology (IRBP) within the Department of Psychology at the University “G. d'Annunzio” of Chieti Pescara (ref: 26005).

### 2.1 Participants

A total of 383 participants completed the survey (Male = 231, Female = 150, non-binary = 2; see Table 1). Participants ranged in age from 11 to 25 years (*M* = 16.50, SD = 2.63) because one of the main goals was to analyze value in adolescent and young adults. Data was collected anonymously, and all individuals participated voluntarily in the study (Table 1). Two non-binary participants were excluded from the analyses because gender was utilized as an independent variable, and the small sample size of this subgroup (*n* = 2) did not provide sufficient statistical power for meaningful group comparisons.

**Table 1:** Participant characteristics across four European countries (Italy, Sweden, Finland, and Poland).

Country	*N*	Female	Age			
			*M*	SD	Min	Max
Italy	149	30	15.60	1.55	13	21
Sweden	79	39	17.10	2.23	14	23
Finland	61	52	17.21	3.76	11	25
Poland	92	29	17.32	3.03	13	25
Total	381	150	16.58	2.65	11	25

### 2.2 Study design

The study followed a cross-sectional comparative design. The independent variables (IVs) were country, gender, age, YOP, and training tier while the dependent variables (DVs) were Moral value, Status value, and Competence value as measured by the YSVQ-2.

### 2.3 Measures

Parallel to the administration of the YSVQ-2 among each country's stakeholders, demographic data (such as age, gender, sport practiced, training tier, and YOP) were collected in the following forms. The country of residency was recorded as Italy, Sweden, Poland, or Finland. The YOP were categorized into three groups: (1) 0–5 years, (2) 6–10 years, and (3) more than 10 years. Age was divided into four categories: (1) under 14, (2) 15–16 years, (3) 17–18 years, and (4) over 19 years old. Lastly, training tiers were classified into three categories based on the participant classification framework by McKay et al. (2022): Tier 1 (“Recreationally Active”), Tier 2 (“Trained/Developmental”), and Tier 3 (“Highly Trained/National Level”; McKay et al., 2022).

### 2.4 YSVQ-2

Lee et al. (2008) developed a 13-item questionnaire, as a modification of the original 18-item YSVQ (Lee et al., 2000). The YSVQ was formatted on single proxy items that represent each value through a personal statement (Lee et al., 2008). The way that YSVQ-2, was reconstructed by Lee et al. (2008) differs from the YSVQ (Lee et al., 2000) by dividing values into three higher-order categories, Moral value, Status value, and Competence value, with multiple items representing each one (Moral = “I try to be fair,” Status = “I win or beat others,” and Competence = “I improve my performance”). In addition, Schwartz's (1992) asymmetric importance scale, originally used and suggested by the questionnaire developers, consists of a seven-item scale from −1 to 5 (from “Opposite of what I believe” to “Extremely important to me”) to measure the importance of the belief an individual assigns to each item (Lee et al., 2008). The present questionnaire is validated by Lee et al. (2008), using confirmatory factor analysis (CFA) to test whether the items fit the three expected value categories (Moral, Competence, and Status). For this study, we adopted Lee et al. (2008) version of the YSVQ-2 keeping the same verbal anchors used in the original scale (“Opposite of what I believe” to “Extremely important to me”) but using a classical Likert scale from 1 to 7 for the scoring to keep consistency within the broader questionnaire of the FAITH project. The scales demonstrated adequate reliability (Cronbach's alpha ranged from 0.60 to 0.80; Table 2). Moreover, an Italian translation of the YSVQ-2 was already used by Danioni et al. (2017), the scale was considered a strong psychometric scale with good cross-cultural validity (Danioni, 2017).

**Table 2:** Reliability of the YSVQ-2 (Cronbach's alpha).

Country	Subscale	Cronbach's alpha	Items	*N*
All	Competence	0.76	4	381
	Moral	0.76	5	381
	Status	0.69	4	381
	Total	0.74	13	381
Italy	Competence	0.75	4	149
	Moral	0.78	5	149
	Status	0.63	4	149
	Total	0.75	13	149
Sweden	Competence	0.72	4	79
	Moral	0.74	5	79
	Status	0.55	4	79
	Total	0.68	13	79
Finland	Competence	0.85	4	61
	Moral	0.77	5	61
	Status	0.69	4	61
	Total	0.83	13	61
Poland	Competence	0.74	4	92
	Moral	0.76	5	92
	Status	0.67	4	92
	Total	0.76	13	92

### 2.5 Data analysis

While overall trends point to consistency in participant responses, several meaningful variations emerge when analyzing estimated marginal means and confidence intervals. These comparisons help clarify whether cultural, developmental, or experiential factors within sport contexts are associated with differences in value endorsement.

To explore cross-national (Country) and individual differences (age, gender, YOP, and training tiers) on the values levels tested with the YSVQ-2, a Multivariate Analysis of Variance (MANOVA) was conducted. The analysis was conducted using IBM SPSS Statistic (27.0). “Other—Gender” was excluded since only two individuals answered it. As independent variables were considered country (Italy, Finland, Sweden and Poland), age (under 14, 15–16 years, 17–18 years, and over 19 years old), YOP (0–5 years, 6–10 years, and more than 10 years), gender (Male and Female) and training tier (Tier 1, Tier 2, and Tier 3). Summarizing we had a MANOVA 2 (gender) × 4 (age categories) × 4 (countries) × 3 (training tiers) × 3 (YOP) on Competence, Status, and Moral Value.

## 3 Results

MANOVA showed a significant effect of “Country,” Wilk's λ = 0.90, *F*_(9, 596.416)_ = 2.798, *p* < 0.05, $\eta_{p}^{2}$ = 0.033; “training tier,” Wilk's λ = 0.931, *F*_(6, 490)_ = 2.98, *p* < 0.05, $\eta_{p}^{2}$ = 0.035, the interaction of “country and training tier,” Wilk's λ = 0.846, *F*_(18, 693.45)_ = 2.338, *p* < 0.05, $\eta_{p}^{2}$ = 0.054 and the interaction of “country, age, and gender,” Wilk's λ = 0.928, *F*_(9, 596.416)_ = 2.074, *p* < 0.05, $\eta_{p}^{2}$ = 0.025. These results show a significant effect of “country,” “training tier,” the interaction of “country and training tier,” and the interaction of “country, age, and gender” on the YSVQ-2 values with a small general effect as shown by the partial eta square.

Following UNIVARIATE showed significant results for “country” on “Status value” *F*_(3, 247)_ = 6.86, *p* < 0.05, $\eta_{p}^{2}$ = 0.077; “training tier” on “Competence value” *F*_(2, 247)_ = 5.75, *p* < 0.05, $\eta_{p}^{2}$ = 0.045, the interaction of “country and training tier” on “Status value,” *F*_(6, 247)_ = 4.17, *p* < 0.05, $\eta_{p}^{2}$ = 0.092 and the interaction of “country, age and gender” on “Moral value,” *F*_(3, 247)_ = 5.028, *p* < 0.05, $\eta_{p}^{2}$ = 0.058. The significance found across all dependent variables (Moral value, Competence value, and Status value) justifies the subsequent *Post-hoc* analysis and the examination of the graphic profiles.

### 3.1 *Post-hoc* analysis

Given the significance of the univariate effects, *post-hoc* comparisons were performed to identify specific differences between group levels, using Bonferroni correction.

#### 3.1.1 Status value

**Figure 1:**
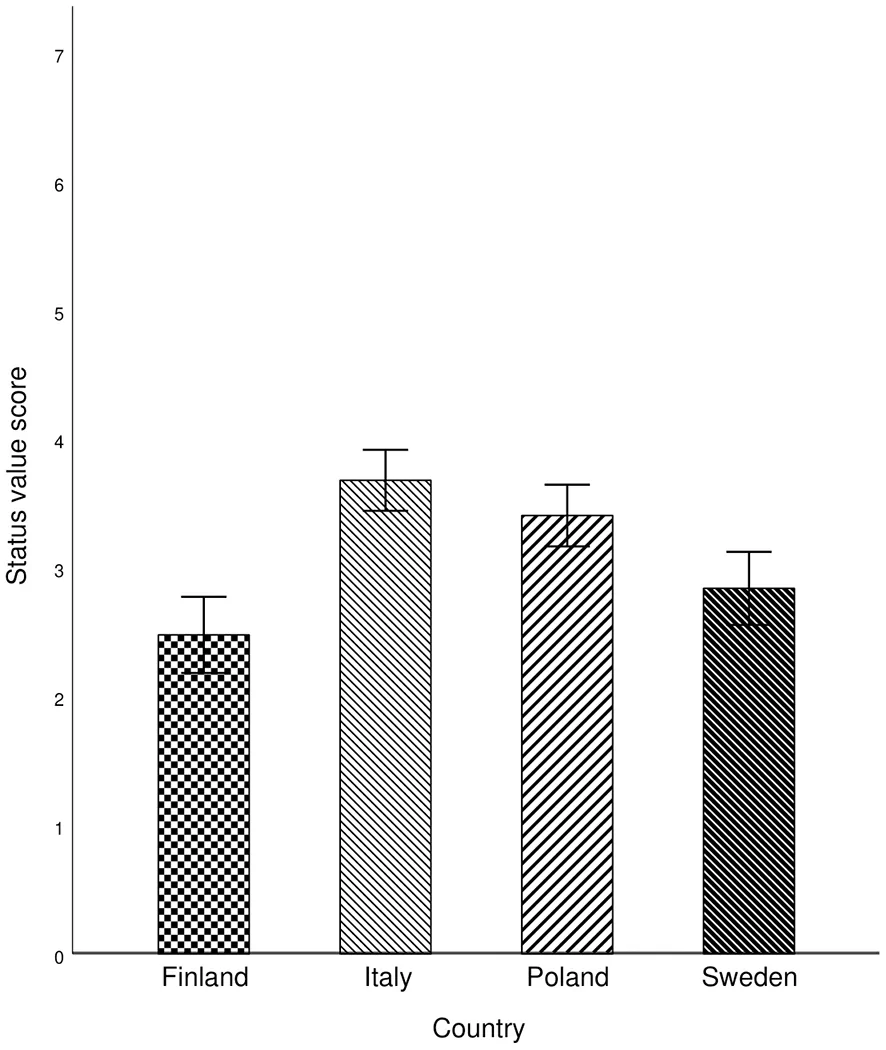
Status value score by Country. Error bars indicate confidence intervals.

Finland results from *Post-hoc* analysis resulted in statistically significant lower scores by Status value when compared to Italy (*p* < 0.05), Poland (*p* < 0.05), and Sweden (*p* < 0.05; Figure 1). Italy resulted in a significantly higher score (*p* < 0.05) when compared with Sweden (Figure 1). Finally, Poland resulted in a significantly higher score (*p* < 0.05) when compared with Sweden (Figure 1). No statistically significant differences have been found comparing the Status value of Italy and Poland.

Moreover, statistically significant differences in Status value were observed when comparing athletes of different tiers across countries. Tier 1 Finnish players scored significantly lower results when compared to Italian, Polish, and Swedish tier 1 athletes (*p* < 0.05). Tier 2 Italian athletes scored statistically significant higher Status value when compared to Swedish, Polish, and Finnish athletes (*p* < 0.05). Finally, Swedish tier 3 athletes resulted in having lower Status value when compared to Finnish, Polish, and Italian players (*p* < 0.05). Other univariate analysis didn't result in a statistically significant result.

#### 3.1.2 Competence value

**Figure 2:**
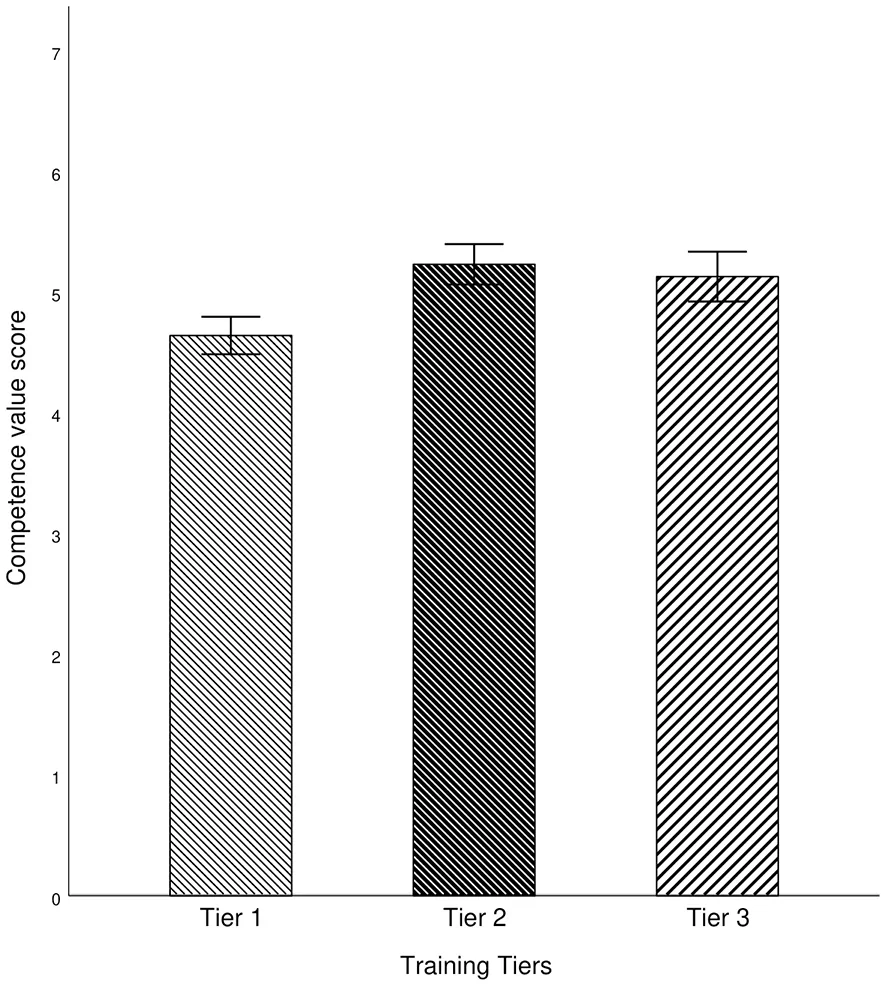
Competence value by training tier. Vertical axis are the scores according to the 0 – 7 likerscale.

Tier 1 athletes' results from *Post-hoc* analysis resulted in statistically significant lower scores by Competence value when compared to tier 2 athletes (*p* < 0.05) and tier 3 athletes (*p* < 0.05; Figure 2). No statistically significant differences have been found comparing Competence value of Elite athletes and Advanced athletes. No other Univariate significant results were found regarding the Competence value. Because Competence represents an individual's self-perceived ability to demonstrate skill, mean differences across performance tiers may be more pronounced than cross-national variations.

#### 3.1.3 Moral value

**Figure 3:**
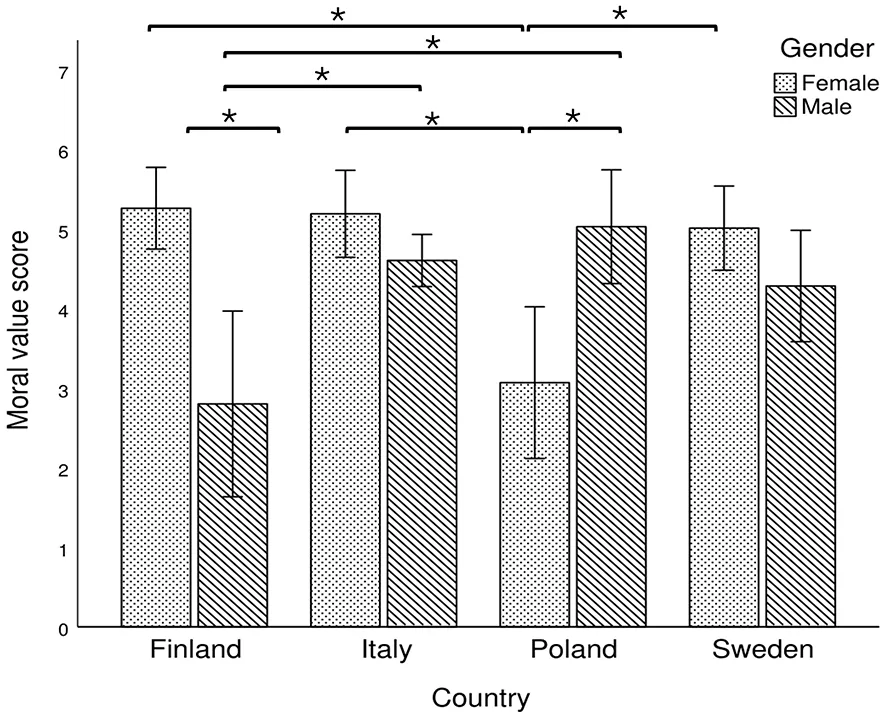
Moral value gender differences by countries in age category 2 (15–16 years). Asterisks indicate statistically significant differences, where *p* < 0.05

A significant three-way interaction between age, gender, and country was found for Moral value. Specifically, 15–16 years old males and females reported significant differences in value scores among the countries. *Post-hoc* comparisons revealed that within age (Category 2 = 15–16 years), Finnish males reported significantly lower Moral value scores compared to their female counterparts of the same age and country (*p* < 0.05; Figure 3). On the other hand, Polish males (Age category 2) reported significantly higher Moral value score compared to the female counterpart (Figure 3). Moreover, comparing countries' female athletes Poland (Age Category 2) scored significantly lower compared to Italy, Finland and Sweden females (Age Category 2). Also, Finnish Males (Age Category 2) scored significantly lower Moral value compared to Italian and Polish Males (Age Category 2). In conclusion, within Age Category 4 (over 19 years), Finnish males scored significantly lower on Moral value relative to same-age females from the same country (*p* < 0.05). No further statistically significant gender differences were observed in any other combination of independent variables.

## 4 Discussion

The main goal of this study was to compare Status, Competence, and Moral value across different countries, age, training tiers, YOP, and gender. YSVQ-2 revealed acceptable reliability in each country's version. The results of the study revealed some notable variations in all the value variables considered in the YSVQ-2.

When examining the Status value, significant variations emerged not only between the national cohorts but also when interacting with athletes' training tier. Specifically, the data indicates a clear geographic trend, with Italian and Polish athletes reporting significantly higher Status value score compared to Finnish and Swedish athletes; notably, the Finnish athletes scored the lowest for this variable. A more nuanced pattern appeared when cross-referencing countries of origin with training tiers. The lower Status value observed in the tier 1 sample appears to be primarily accounted for by the Finnish subgroup compared to their foreign peers. On the other hand, tier 2 Italian athletes scored higher Status value compared to the same tier foreign athletes. Finally, tier 3 Swedish athletes reported significantly lower Status value when compared to the corresponding tier groups of the other examined countries.

Regarding competence values, a significant main effect emerged when comparing the participants' training tiers. Our findings indicate that tier 1 athletes reported significantly lower competence value scores than both tier 2 and tier 3 athletes. Moreover, no statistically significant difference was observed between the advanced and elite performance groups.

Finally, a significant interaction was found for Moral value when cross-referencing gender, country, and age. Specifically, among youth cohorts, young Finnish male athletes reported significantly lower moral scores compared to their female age peers. In contrast, within the same youth category, Polish male athletes exhibited significantly higher Moral value than Polish female athletes. Lastly, when examining the age category 4 demographic (aged 19 and above), Finnish males demonstrated significantly lower Moral value relative to same-age females.

The prominent cross-national variations observed in Status value may fundamentally reflect structural diversities in the respective sport systems. Specifically, Northern European countries such as Finland and Sweden share deeply rooted commonalities regarding their cultural frameworks, socio-economic development, and political systems (Bergsgard et al., 2019). The Italian sport system is co-managed by the Italian government through in-house society of the Ministry of Economy, “Sport e Salute Spa.” and the Italian Olympic Committee (CONI). “Sport e Salute” is an Italian company renamed in 2019, from “CONI Servizi Spa” 2002.The main goal of “Sport e Salute Spa” is to adopt sport as a connection between communities, with a remark of the inclusion of all individuals. On the other hand, the Finnish sporting model is highly institutionalized and strictly regulated by legislative frameworks, most notably the landmark Sports Act of 1978 (Bergsgard et al., 2019). This legislation conceptualizes sporting practices comprehensively, encompassing grassroots physical activity, elite-level competition, and civic engagements, while placing a paramount emphasis on public health and holistic wellbeing (Bergsgard et al., 2019). Local municipalities are legally tasked with the direct governance and operational management of sporting activities, utilizing dedicated state grants to sustain local programs (Bergsgard et al., 2019). In contrast, the Swedish model exhibits a more decentralized governance structure. While funding is centrally administered by the Swedish Sports Confederation (*Riksidrottsförbundet*), individual sporting organizations retain a high degree of autonomy in managing and allocating their respective financial resources (Bergsgard et al., 2019). Consequently, the Swedish national government maintains a less interventionist and more indirect role relative to its Finnish counterpart; nevertheless, its systemic influence remains substantial, particularly through the funding and regulation of the vast majority (57%) of the country's sports facilities (Bergsgard et al., 2019). Taken together, despite these minor governance differences between the two Nordic nations, both systems share a foundational commitment to democratic access, public welfare, and operational decentralization. This structural organization might prioritize personal growth and community cohesion over commercial or result-oriented pressures. On the other hand, Southern European nations often display distinct socio-cultural configurations, where social life and core values tend to be anchored around familial structures and close relational networks, rather than a predominantly civic-oriented one (Bergsgard, 2025). These socio-cultural differences offer an explanation for the significant discrepancies in Status scores between the Italian and Finnish cohorts. Specifically, the contrast between a highly institutionalized, welfare-oriented civic system and a culture more closely aligned with relational prestige and competitive achievement may provide a descriptive contextual background for the observed subgroup differences, where elevated Status value endorsement among Italian athletes contrasts with the lower scores found in the Finnish sample. For example, Italian adolescents have been shown to maintain a high level of commitment toward an athletic career, despite acknowledging its uncertain and limited prospects (Guidotti et al., 2013); in such precarious contexts, achieving elite athletic success may become a primary vehicle for personal prestige and social validation rather than a structured career path. Differences in national sports governance offer a contextual lens for understanding how value endorsement varies among young athletes across European cohorts, though intermediate factors (i.e., coaching practices and team environment) need further investigation. Economic necessity might push sport organizations to focus more on results and be more competitive, rather than promote the positive aspects of sport such as health benefits, socialization and adolescents' personal growth. Values reflect future behaviors and since young athletes represent the future generation, it's important to avoid promoting activities that might lead to antisocial behaviors in the sport context.

Given that competence values are generally associated with prosocial outcomes (Lee et al., 2008), examining potential variations across different athletes' tiers is essential to determine whether this relationship remains consistent from recreational to elite sports settings. Interestingly, literature shows that while amateur athletes align more closely with fair play, elite and individual competitors exhibit a higher tendency to sacrifice moral principles for victory (Barcsi and Filò, 2023). Moreover, as demonstrated by Mallia et al. (2019) in high-level sports, satisfying the need for competence (as psychological construct) often couples with controlled motivation (Mallia et al., 2019). Under these external pressures, athletes struggle to “keep winning in proportion” fostering an “antisocial attitude” where cheating and gamesmanship are accepted as functional, tactical strategies to secure victory (Mallia et al., 2019). This aligns with Sage et al. (2006) who discussed that a strong ego orientation athlete may define success by “outperforming opponents” with a general “preoccupation with winning” (Sage et al., 2006). Crucially, high levels of ego orientation cover up the protective effect of task goals on moral variables, making antisocial behavior acceptable if deemed necessary to win (Sage et al., 2006). Consequently, higher tier players may perceive unsportsmanlike behavior as a necessary, functional tool to secure victory when the stakes of the competition are elevated.

Furthermore, cross-cultural gender differences in moral judgment were documented by Atari et al. (2020) in a large-scale study spanning 67 countries (Atari et al., 2020). Their findings revealed that females consistently scored higher than males in the dimensions of Care, Fairness, and Purity. These dimensions are included in the “Moral Foundation Theory” proposed by (Graham et al., 2013), other dimensions included in the model are “Loyalty” and “Authority” (Graham et al., 2013). This moral gap expands in individualistic and egalitarian societies but shrinks in male-biased cultures where men place a greater emphasis on family and parenting (Atari et al., 2020). The reversed pattern observed among Polish youth may reflect a more traditional cultural context, where male social roles are more strongly tied to group loyalty, potentially elevating moral value endorsement among young male athletes.

The studies of (Mallia et al., 2019; Atari et al., 2020) and (Sage et al., 2006) offer important insights into how competitive levels and gender relate to athletic attitudes and gender as two major factors associated with differences in athletic behavior. Although our study advanced the knowledge in the field and explored value differences across European countries, more investigation is needed to better understand the complex relationship underlying the differences in sport values. Additionally, Status, Moral, and Competence are crucial psychological factors associated with athletes' attitudes and conduct. Gaining a deeper comprehension of these values can significantly enhance our understanding of value endorsement patterns across different national subgroups, coaches should consider value differences in young athletes in order to create more country specific trainings. Several methodological limitations of the present study should be acknowledged. First, the recruitment strategy relied on convenience sampling through project workshops rather than randomized probability sampling, which may constrain the generalizability of the findings. Second, the sample exhibited an uneven gender distribution across national cohorts and lacked representation of non-binary or gender-diverse participants.

## 5 Conclusions

Our study highlights the importance of understanding national differences in values among young athletes. The findings suggest that cultural contexts are associated with distinct value endorsement profiles in the sport context, highlighting the context-relation aspect of values. Coaches and sport structures should take into account these differences. In particular, developing country-specific and context-aware trainings could better support positive value development among athletes. Further research could focus on how a specific program is developed in the framework of the FAITH project and how coaches can influence the process.

Finally, since this study adopted a cross-national perspective, future research should focus on more in-depth, country-specific analyses to better understand how values are expressed within each unique sporting environment and how coaches can support the value transmission process.

## Data Availability

The raw data supporting the conclusions of this article will be made available by the authors, without undue reservation.
